# Semi-quantitative FDG-PET Analysis Increases the Sensitivity Compared With Visual Analysis in the Diagnosis of Autoimmune Encephalitis

**DOI:** 10.3389/fneur.2019.00576

**Published:** 2019-06-06

**Authors:** Rui-Juan Lv, Jian Pan, Guifei Zhou, Qun Wang, Xiao-Qiu Shao, Xiao-Bin Zhao, Jiangang Liu

**Affiliations:** ^1^Department of Neurology, Beijing Tiantan Hospital, China National Clinical Research Center for Neurological Diseases, Capital Medical University, Beijing, China; ^2^School of Computer and Information Technology, Beijing Jiaotong University, Beijing, China; ^3^Department of Nuclear Medicine, Beijing Tiantan Hospital, Capital Medical University, Beijing, China; ^4^Beijing Advanced Innovation Center for Big Data-Based Precision Medicine, School of Medicine, Beihang University, Beijing, China

**Keywords:** autoimmune encephalitis, FDG-PET, semi-quantitative analysis, visual analysis, sensitivity

## Abstract

**Objective:** The purpose of this study is to evaluate the potential diagnostic benefit of SPM-based semi-quantitative FDG-PET analysis in autoimmune encephalitis (AE) compared with visual analysis by experienced neuroradiologists using a larger sample size.

**Methods:** This observational retrospective case series study was conducted from a tertiary epilepsy center between May 2014 and March 2017. Healthy individuals without any neurologic or psychiatric diseases were recruited as control. We determined brain FDG-PET abnormal glucose metabolism on medial temporal lobe and basal ganglia using semi-quantitative analysis and compared this method with visual analysis at the same time among patients with autoantibody positive AE.

**Results:** Twenty-eight patients with clinically diagnosed AE and 53 healthy individuals without any neurologic or psychiatric diseases were recruited. On the medial temporal lobe and the basal ganglia, semi-quantitative analysis showed consistency with the visual assessment for whom they had abnormal metabolism by visual assessment. More importantly, 56% patients on medial temporal lobe and 73% patients on the basal ganglia respectively who were not identified by visual inspection can be detected by semi-quantitative analysis, demonstrating the greater sensitivity of semi-quantitative analysis compared with visual assessment.

**Significance:** This study showed semi-quantitative brain FDG-PET analysis was better than visual analysis in view of observing the abnormal glucose metabolism of patients with autoantibody positive AE. Semi-quantitative FDG-PET analysis appears to be a helpful tool in early diagnosis of patients with AE.

## Introduction

The clinical diagnosis of autoimmune encephalitis (AE) can be difficult since patients manifest various unspecific symptoms that overlap with other encephalitis such as infectious encephalitis (1). The clinical symptoms are varied and include seizures, rapid cognitive decline, behavioral problems, and so on. The diagnosis can be made according to clinical manifestation and serum and/or cerebrospinal fluid (CSF) autoantibody test. Autoantibody testing is not readily available at many institutions for technical reasons. Even if it is available at some institutions, it will take several weeks to obtain (1). In addition, failure to detect a neural antibody cannot exclude AE when other clinical clues exist (2, 3). The early diagnosis of AE is very important because early immunotherapy may slow, halt, or even reverse the disease process (2, 4, 5), whereas delaying initiation of immunosuppressive therapy may result in serious disability, or even death if left untreated (6). Depending on autoantibody testing to make the diagnosis may significantly delay treatment initiation. Therefore, early diagnosis should not be delayed because of awaiting for serological or CSF autoantibody result (1). Early neuroimaging, as well as the initial neurological assessment, plays a key role in the early diagnosis of AE (1, 7).

Recently, leading authorities on the diagnosis and treatment of AE convened and recommended criteria for the diagnoses of possible, probable and definite AE to mitigate the delay in initiation of therapy while awaiting autoantibody assay results (1). Francesc Graus et al. proposed the diagnosis of AE was made according to the neurological evaluation and the corresponding diagnostic correlated examinations including magnetic resonance imaging (MRI), CSF and serum sampling, as well as electroencephalography (EEG) results (1, 8). Prompt diagnosis often relies on neuroimaging. In terms of brain imaging, the recommended diagnostic framework frequently only depends on cranial MRI. Nevertheless, a relevant proportion of AE patients had normal or non-specific MRI results (8, 9). Therefore, the normal MRI finding does not exclude an immune-mediated process. In AE, 2-deoxy-2-18F fluoro-D-glucose-(18F-FDG) positron emission tomography/computed tomography (PET/CT) imaging has been reported to representatively show hypermetabolism on the medial temporal lobe in MRI-negative patients, suggesting that it is better than MRI in the diagnostic framework (8–10).

Traditionally, whole body 18F-FDG-PET has been utilized to assess for occult malignancy as a cause for AE. Recent publications have begun to explore the added value of brain 18F-FDG PET/CT in evaluating these patients (11–13). Several groups have suggested that 18F-FDG PET/CT may be used to evaluate efficacy of therapy or monitor the suspected disease recurrence (11, 14). FDG-PET appears to be a helpful tool in early diagnosis of patients with AE, especially those with normal MRI scans. Some researchers have tried to use 18F-FDG PET/CT to perform early diagnosis of suspicious AE before acquiring the autoantibody results (8). However, the further studies are needed to verify its predictive value for the early diagnosis of AE, and the larger validation studies are needed (15). Therefore, we would like to ulteriorly find the major reliable evidence on the underlying role of 18F-FDG PET for the earlier diagnosis of AE.

Despite visual analysis is often the first step of brain 18F-FDG PET reading, the standardization in view of reading images and reporting results is defective now (16). In clinical practice, the lack of expertise and objective semi-quantitative detection methods will hold back the comprehension of the real disease pattern and lead to misinterpreted report. Some automated approaches to analyze 18F-FDG PET data are not applicable for finding the hypermetabolism in AE, since they were developed to diagnose the hypometabolic patterns in Alzheimer's disease (17). In contrast, some SPM-based semi-quantitative measures might protrude the areas of relative hypermetabolism owing to the bias introduced by intensity normalization procedures (18). Moreover, one recent report showed that semi-quantitative analysis can reveal subtle changes, suggesting that semi-quantitative analysis was better than qualitative reporting (19).

In the present study, we modified the semi-quantitative FDG-PET analysis as a biomarker for definite, autoantibody positive AE, allowing for additional region of interest (ROI) analyses of the mesial temporal lobe and basal ganglia. We used this analysis method in patients with antibody positive AE. The aim was to evaluate the potential diagnostic value of this analysis method compared with visual analysis by experienced neuroradiologists.

## Subjects and Methods

### Participants

We recruited 28 AE patients with autoantibody (56 ± 11 years; 22 males) in our Tertiary Epilepsy Center from May 2014 to April 2017. All patients who had undergone cerebral FDG-PET were included in this retrospective study. Fifty-three healthy individuals (52 ± 7 years; 31 males) without any neurologic or psychiatric diseases were recruited. The age of these two group of participants were well matched and showed no significant difference [*t*_(79)_ = 1.97; *p* > 0.05). The detailed demographics of participants were presented in Table 1. Written Informed consent to participate the study and for publication for clinical details were obtained from each subject enrolled. The study was approved by the Medical Ethics Committee of Beijing Tiantan Hospital, Capital Medical University and was carried out in accordance with the Declaration of Helsinki.

**Table 1:** Demographics of participants.

**Group**	**Age (years)**		**Gender**	
	**Range**	**Mean ± SD**	**Male**	**Female**
AE patients (*n* = 28)	34~78	56.32 ± 10.93	22	6
Healthy individuals (*n* = 53)	59~69	52.47 ± 6.66	31	22

Cases of AE included in the study were patients presenting with new onset electrographic seizure activity, plus at least two of the following: (1) CSF findings consistent with inflammation [elevated CSF protein >45 mg/dl and/or lymphocytic pleocytosis; elevated CSF immunoglobulin G (IgG) index and/or positive oligoclonal bands (OB)]; (2) brain MRI or FDG-PET showing signal changes consistent with limbic encephalitis; (3) autoimmune/paraneoplastic antibodies in serum and/or CSF which have been associated with autoimmune encephalitis in previous studies (any neuronal nuclear/cytoplasmic antibody such as anti-Hu, Yo, Ri, Ma2/Ta, CV2/CRMP5, amphiphysin; any neuronal membrane antibody including anti-NMDA-R, CASPR2, AMPA1-R, AMPA2-R, LGI1, and GABA_B_-R antibody), (4) new onset seizure responding to immunomodulatory therapies. Cases were excluded if there was evidence of another identified cause of the patient's seizures: (1) presence of CSF viral/bacterial/fungal antigens or antibodies or DNA PCR which could explain underlying acute inflammatory brain parenchymal changes, (2) presence of metabolic abnormalities which could have precipitated seizures (severe renal or hepatic failure, malignant hypertension, severe hypo/hyperglycemia), (3) presence of brain structural lesions such as stroke, tumor, traumatic lesions, heterotopias, vascular malformation, abscess or infectious lesion which could have precipitated the presenting seizures.

### Neuronal Antibody Measurement

AE in the present study were definitely diagnosed by autoantibody assay. All suspected AE patients underwent serum and CSF antibody test. Serum and CSF samples had been sent for antibody test to the laboratory of neurological immunology of Peking Union Medical College Hospital. Serum and CSF titers for onconeural antibodies anti-Hu, Yo, Ri, CV2/CRMP5, amphiphysin, Ma2/Ta, and the neuronal surface antibodies anti-NMDA-R, CASPR2, AMPA1-R, AMPA2-R, LGI1, and GABA_B_-R were measured with both cell-based assay and immunohistochemistry in serum and CSF.

### Cerebral Imaging Acquisition

The brain ^18^F-FDG PET/CT scan was performed to evaluate the glucose metabolism of each participant. All participants were fasted for at least 6 h and their blood glucose levels were confirmed to be within the normal range before injection of ^18^F-FDG. The subjects were injected with 0.10–0.15 mCi/kg of ^18^F-FDG. Then, after 30 min rest in a dimly lit room, they underwent the brain PET/CT scans (eyes open, reduced ambient noise). PET/CT images were acquired with the use of a multidetector helical PET/CT scanner (Discovery 690, GE Medical Systems). All cerebral FDG-PET studies were done in conjunction with whole-body PET scans (in search of malignancies; brain scan first).

### Analysis

#### Visual Assessment

Previous study demonstrated that AE were usually associated with the abnormalities of glucose metabolism in some brain regions, such as medial temporal lobe and basal ganglia (1). Thus, to assess the glucose metabolism in these brain areas, the PET images of each patient were visually examined by three reviewers independently. One of them was an attending doctor of nuclear medicine (X-B Zhao) with vast experience of reading PET/CT (>10 years) and the other two were experienced neurology specialists [R-J Lv (9 years) and X-Q Shao (15 years)]. Three reviewers were asked to carefully diagnose whether the medial temporal lobe and basal ganglia showed abnormal glucose metabolism or not. In addition, they would assess the lateralization of the abnormality when it existed. All reviewers were blinded from clinical diagnosis of the conditions of either cases or controls. These three specialists discussed together to reach a consensus when their original evaluations were discordant. The Kappa coefficient of three specialists was 0.86.

#### Semi-quantitative Analysis

##### Image preprocess

The brain PET scans were also assessed through semi-quantitative analysis. The analysis including preprocessing and statistical analysis, were mainly implemented using the MATLAB and Statistical Parametric Mapping software (SPM12, Wellcome Trust Center for Neuroimaging, London, UK; https://www.fil.ion.ucl.ac.uk/spm/software/spm12/). During the preprocessing, the co-registration between PET metabolism images and CT structural images was firstly performed. Then, the CT images with high resolution were spatial normalized into Montreal Neurological Institute template (MNI). Thus, the PET images were also normalized into this standard templates using the computed space transformation for CT normalization. Finally, the PET scans were resampled to 2 × 2 × 2 mm^3^ voxels and spatially smoothed using an isotropic 6 mm full-width-half-maximal (FWHM). Additionally, to remove the bias of global metabolism, each voxel's intensity of PET scans was normalized by dividing the average of the voxels within the highest 20% of intensity (20).

##### Statistical analysis

**Figure 1:**
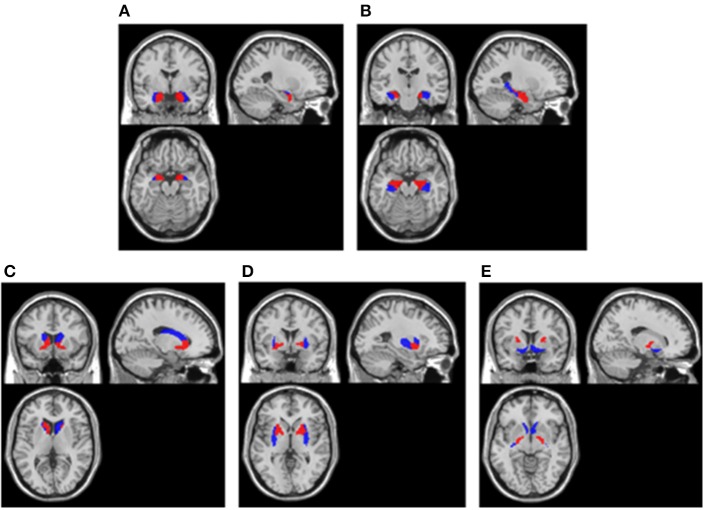
ROIs extracted in cerebral cortex. **(A)** Amygdala. Blue areas were lateral amygdala and red areas were medial amygdala; **(B)** Hippocampus. Blue areas were caudal hippocampus and red areas were rostral hippocampus; **(C)** Caudate nucleus. Blue areas were dorsolateral caudate nucleus and red areas were ventral caudate nucleus; **(D)** Putamen. Blue areas were dorsolateral putamen and red areas were ventromedial putamen; **(E)** Pallidum and Nucleus accumbens. Blue areas were nucleus accumbens and red areas were pallidum. All ROIs were extracted using the Human Brainnetome Atlas (21) and they were overlay on a MNI single subject T1 scan (“/spm12/canonical/single_subj_T1.nii”).

In line with the above visual assessment, we also investigated the metabolism of brain regions located in medial temporal lobe and basal ganglia using statistical analysis. Considering the size bias of the abnormalities in these areas, the present study not only examined the entire medial temporal lobe and basal ganglia, but also examined their fine-grained divisions. Using the Human Brainnetome Atlas (21), we defined the following regions of interest (ROIs) within medial temporal lobe: (1) bilateral medial amygdala, (2) bilateral lateral amygdala, (3) bilateral caudal hippocampus, (4) bilateral rostral hippocampus, and (5) entire bilateral medial temporal lobe consisting of 1–4; within basal ganglia: (6) bilateral dorsolateral caudate nucleus, (7) bilateral ventral caudate nucleus, (8) bilateral dorsolateral putamen, (9) bilateral ventromedial putamen, (10) bilateral pallidum, (11) bilateral nucleus accumbens, and (12) entire bilateral basal ganglia consisting of 6–11. The locations of these ROIs in MNI template showed in Figure 1.

For each ROI within medial temporal lobe, the z-score was calculated as an indicator of abnormalities of glucose metabolism of each patient deviated from that of the control healthy group. Specifically, we firstly calculated the mean of whole voxels' intensities within each ROI to obtain the average metabolic of the ROI for each participant. Then, for each ROI, we calculated the mean (M) and standard deviation (SD) of the average metabolic cross all healthy individuals. Finally, for each patient, z-score was obtained by subtracting M from each ROI and then dividing the SD [namely, z-score = (patient-M healthy subjects)/SD healthy subjects]. The medial temporal lobe were identified hyper-metabolism if any of ROIs within the medial temporal lobe showed a significant higher metabolism than healthy group (z > 1.96, *p* < 0.05). Whereas, the medial temporal lobe were identified hypo-metabolism if any of ROIs within the medial temporal lobe showed a significant lower metabolism than healthy group (*z* < −1.96, *p* < 0.05). For each ROI within basal ganglia, the same statistical analysis as the ROIs within medial temporal lobe was conducted on each patient.

## Results

### Autoantibody Assay

The outcome of autoimmune antibody subtypes of each patient was showed in Table 2. Twenty-three patients were diagnosed as anti-LGI1 encephalitis, and four patients were diagnosed as anti-GABA_B_R encephalitis, and the other one was diagnosed as anti-amphiphysin encephalitis.

**Table 2:** Results of duration, autoantibody assay, visual assessment and semi-quantitative analysis.

**Patient no**.	**Duration (month)**	**Autoantibody assay**	**Medial temporal lobe**		**Basal ganglia**	
			**Visual assessment**	**Semi-quantitative analysis**	**Visual assessment**	**Semi-quantitative analysis**
1	6	Amphiphysin	NO	Left	NO	NO
2	1	GABA_B_-R	NO	Bilateral	NO	Bilateral
3	1	GABA_B_-R	Bilateral (R)	Bilateral	NO	Bilateral
4	1	GABA_B_-R	Bilateral (R)	Bilateral	NO	Bilateral
5	8	GABA_B_-R	NO	NO	NO	Bilateral^*^
6	2	LGI1	NO	Left^*^ and Right	NO	Bilateral
7	0.5	LGI1	Bilateral (R)	Bilateral	Bilateral	Bilateral
8	0.5	LGI1	NO	Bilateral	Bilateral (R)	Bilateral
9	4	LGI1	Bilateral (L)	Bilateral	Bilateral (L)	Bilateral
10	3	LGI1	Bilateral (R)	Bilateral	Bilateral (L)	Bilateral
11	1	LGI1	Bilateral (R)	Bilateral	Bilateral	Bilateral
12	2	LGI1	Bilateral (R)	Bilateral	Bilateral (R)	Bilateral
13	10	LGI1	NO	NO	NO	NO
14	6	LGI1	Bilateral (L)	Left	NO	NO
15	6	LGI1	NO	NO	Bilateral	Bilateral
16	7	LGI1	NO	NO	NO	Right
17	3	LGI1	NO	Bilateral	NO	Left^*^ and Right
18	3	LGI1	Bilateral (L)	Bilateral	NO	Left
19	3	LGI1	Bilateral	Bilateral	Bilateral (L)	Bilateral
20	1.5	LGI1	Bilateral (L)	Bilateral	Bilateral (R)	Bilateral
21	2	LGI1	Bilateral (R)	Bilateral	Bilateral (R)	Bilateral
22	2	LGI1	Bilateral (L)	Left	Left	Left and Right^*^
23	6	LGI1	Bilateral (R)	Bilateral	Bilateral (L)	Bilateral
24	2	LGI1	Bilateral (L)	Bilateral	Bilateral (L)	Bilateral
25	0.5	LGI1	Bilateral (L)	Bilateral	Bilateral	Bilateral
26	3	LGI1	Bilateral (L)	Bilateral	Bilateral (L)	Bilateral
27	1	LGI1	Bilateral (R)	Bilateral	Bilateral	Bilateral
28	1	LGI1	Bilateral (L)	Bilateral	Bilateral (L)	Bilateral

* *The ROI was identified as hypometabolism*.

*Bilateral (L/R) means that the level of hypermetabolism in left/right regions was higher than right/left regions*.

### Visual Assessment Results

The visual assessment results were shown in Table 2. On medial temporal lobe, 19 patients were verified as hyper-metabolism. However, the visual assessment failed to identify the other nine patients. Thus, the sensitivity of visual assessment for autoimmune encephalitis on medial temporal lobe was about 68% (19/28). One patient among the 19 patients successfully detected by visual assessment showed a comparable hyper-metabolism between the right hemisphere and the left hemisphere. However, nine patients showed worse abnormalities of glucose metabolism in the right hemisphere than the left hemisphere, while the other nine patients showed worse abnormalities of glucose metabolism in the left hemisphere than the right hemisphere.

On basal ganglia, 17 patients were identified as hyper-metabolism through visual assessment. However, the other 11 patients had been missed. Thus, the sensitivity of visual assessment on medial temporal lobe was about 61% (17/28). Five patients among the 17 patients successfully detected by visual assessment showed a comparable hyper-metabolism between the right hemisphere and the left hemisphere. However, four patients showed worse abnormalities of glucose metabolism in the right hemisphere than the left hemisphere, while the other seven patients showed worse abnormalities of glucose metabolism in the left hemisphere than the right hemisphere. Besides, one patient among the 17 patients showed the hyper-metabolism only in the left hemisphere.

### Semi-quantitative Analysis Results

The results of semi-quantitative analysis were also shown in Table 2. On medial temporal lobe, 24 patients were identified as abnormal glucose metabolism. However, in the other four patients, the PET images didn't detect any abnormality. Thus, the sensitivity of semi-quantitative analysis on medial temporal lobe was about 86% (24/28).

On basal ganglia, 25 patients were identified as abnormal glucose metabolism. However, the other three patients weren't identified as abnormality. Thus, the sensitivity of semi-quantitative analysis on basal ganglia was about 89% (25/28).

### Comparison Between Semi-quantitative Analysis and Visual Assessment

On the medial temporal lobe, as shown in Table 2, 19 patients were identified as bilateral hyper-metabolism by visual assessment. All these patients were also successfully identified hyper-metabolism through semi-quantitative analysis, showing consistency with the visual assessment. The Kappa coefficient between visual and SPM analysis was 0.82. In addition, the lateralization of results from both analyses seemed no difference except two subjects. Subject 14 and 22 who were identified as bilateral hyper-metabolism on visual assessment were found only left medial temporal lobe hyper-metabolism by semi-quantitative analysis.

More importantly, five of nine patients who were not identified by visual inspection (56%) were detected by semi-quantitative analysis, demonstrating the greater sensitivity of semi-quantitative analysis compared with visual assessment. Three of these five patients were identified as a bilateral abnormality and one patient was found to be abnormal only on left medial temporal lobe, and the other patient was found hyper-metabolism on the right medial temporal lobe while hypo-metabolism on the left medial temporal lobe.

On the basal ganglia, as shown in Table 2, 17 patients were identified as bilateral hyper-metabolism by visual assessment. All of them were also identified hyper-metabolism through semi-quantitative analysis, showing consistency with the visual assessment. The Kappa coefficient between visual and SPM analysis was 0.71. The lateralization of results from both analysis seemed no difference. In addition, subject 22 who were identified as hyper-metabolism only on the left basal ganglia through visual assessment was found hyper-metabolism on the left basal ganglia while hypo-metabolism on the right basal ganglia through semi-quantitative analysis.

More importantly, eight of 11 patients not identified by visual inspection (73%) were detected by semi-quantitative analysis, demonstrating the greater sensitivity of semi-quantitative analysis compared with visual assessment. Four of these eight patients were identified as bilateral hyper-metabolism, and Four of these eight patients were identified as bilateral hyper-metabolism, and one patient (subject 18) was found hyper-metabolism only on the left basal ganglia, and one patient (subject 16) was found hyper-metabolism only on the right basal ganglia, and one patient (subject 17) was found hyper-metabolism on the right basal ganglia while hypo-metabolism on the left basal ganglia, and the other one patient (subject 5) was found hypo-metabolism on the bilateral basal ganglia.

**Figure 2:**
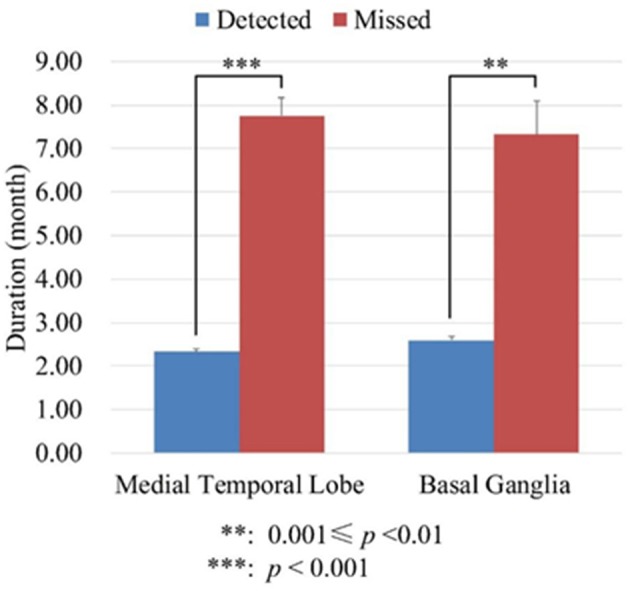
The durations of disease (month) of detected and missed group patients. The detected group meant in these patients abnormal metabolism can be detected by semi-quantitative analysis. The missed group meant in these patients abnormal metabolism cannot be detected by semi-quantitative analysis.

Although the sensitivity of semi-quantitative analysis increased significantly compared with visual inspection, it cannot detect abnormal metabolism in all those patients without abnormal metabolism by visual inspection. To know if disease duration was the interference factor, we divided the patients into two groups: in one group of patients abnormal metabolism can be detected (detected group) by semi-quantitative analysis; in the other group of patients abnormal metabolism cannot be detected (missed group) by semi-quantitative analysis. We analyzed the disease duration of the two groups of patients using the independent *t*-test. On the medial temporal lobe, the mean duration of detected group was 2.33 ± 1.71 months, whereas the mean duration of missed group was 7.75 ± 1.71 months. On the basal ganglia, the mean duration of detected group was 2.60 ± 2.10 months, whereas the mean duration of missed group was 7.33 ± 2.31 months. These results suggested that the patients without abnormal metabolism through semi-quantitative analysis had significantly longer disease duration (Seen in Figure 2).

## Discussion and Conclusion

To date, FDG-PET/CT is one of largest increases in the numbers of medical imaging scanners (22). Moreover, FDG-PET/CT has been considered to be better than other conventional imaging tests in the clinical diagnostical settings, and it has showed good cost-effectiveness for non-small lung cancer staging (22). In addition, FDG-PET plays a key role in view of screening for occult malignancy for patients with paraneoplastic syndromes, including AE (23). Therefore, FDG-PET will probably become more commonly used inspection method for evaluating the suspicious AE patients in addition to malignancy screening (24). Many institutions apply a “vertex to toe” whole-body screening for malignancy. The additional 10 min dedicated brain 3D PET acquisition does not require extra radiopharmaceutical administration, which is easily brought into routine clinical workflows (24).

Since early immunotherapy can lead to better prognosis in AE, recent criteria have been improved to get early diagnosis (1). FDG-PET has been proposed to be a potentially useful diagnostic neuroimaging marker in suspected AE (8, 25). Some AE patients had normal mesial temporal lobe structures on MRI, whereas they showed hypermetabolism on FDG-PET (9, 26). These suggest that FDG-PET is more important than MRI in the early diagnosis and prognosis improvement of AE. Most previous studies of FDG-PET in AE only qualitatively described the FDG-PET findings (27–29). However, to date, there was lack of standard procedures for reading of FDG-PET imaging data in AE. Therefore, we sought to discuss the potential diagnostic benefit of semi-quantitative FDG-PET analysis compared with visual analysis by experienced neuroradiologists.

In the present study, we described semi-quantitative brain FDG-PET findings among patients with autoantibody positive AE, and compared this method with visual analysis at the same time. Our results showed that semi-quantitative brain FDG-PET analysis can find abnormal metabolism more sensitively, suggesting that semi-quantitative brain FDG-PET analysis was better than visual analysis. This study included a larger proportion of patients with LGI1. Previous case studies found striatal hyper-metabolism and/or the medial temporal lobe hyper-metabolism in patients with LGI1 encephalitis (1). However, the majority of these studies were described based on qualitative analyses only. Visual analysis is subjective and dependent on expertise, the level of experience can increase diagnostic accuracy of correlation with the clinical diagnosis, however, this effect was not affected using the analysis of SPM images (30, 31). Accurate visual analysis requires a good knowledge of normal distribution of F-18 FDG in various ages, characteristic distribution of metabolic abnormalities in various subtypes of AE, and normal brain anatomy and recognizing abnormal findings on low-dose CT scan and certain artifacts on PET/CT images. In the field of research in dementia, various semi-quantitative analysis programs have been developed over the years to detect mild abnormalities which are not apparent on visual inspection (32–34). Similar to dementia, it is urgent to develop various semi-quantitative analysis programs in AE. For the first time, we describe brain FDG-PET findings using semi-quantitative analysis among patients with autoantibody AE using a larger sample size.

The striatal hyper-metabolism in voltage-gated potassium channel-complex (VGKCc) encephalitis has been described previously (4, 35, 36) and may highly suggested the patients maybe positive for VGKCc antibodies, particularly in those patients with faciobrachial dystonic seizures (4). Faciobrachial dystonic seizures seem to strongly linked to the leucine-rich glioma inactivated-1 (LGI1) protein target of VGKCc antibodies. Besides, hippocampus is the area where LGI1 protein most strongly expressed (37) superior to the striatum. Anti-GABA_B_R and anti-amphiphysin encephalitis as well as anti-LGI1 encephalitis all belong to limbic encephalitis. In addition, our previous study also showed that anti-GABA_B_R encephalitis and anti-amphiphysin encephalitis had similar clinical manifestation to anti-LGI1 encephalitis (3). Therefore, we chose medial temporal lobe and basal ganglia as ROI to observe the abnormalities of glucose metabolism. By our clinical observation, we found amygdala hyper-metabolism is more common in patients with LGI1. Besides, considering the size bias of the abnormalities in these areas, the present study not only examined the entire medial temporal lobe and basal ganglia, but also examined their fine-grained divisions. The fine-grained divisions can increase the sensitivity of finding abnormal metabolism. For example subject 2, we cannot detect abnormal glucose metabolism in entire medial temporal lobe, however, we can detect hyper-metabolism in right medial amygdala, bilateral lateral amygdala, and right rostral hippocampus.

Although semi-quantitative analysis can detect abnormal metabolism more sensitively compared with visual analysis, the positive rate can only reach 50% on medial temporal lobe and 67% on basal ganglia, respectively. We suppose that is due to the different disease duration. Previous studies showed that FDG-PET abnormal metabolism can change with disease evolution. Intense 18F-FDG uptake can be found in bilateral limbic system at active disease status, and then the 18F-FDG uptake decreased gradually and eventually returned to normal following the clinical improvement after treatment (12, 13). In addition, one study in regard to progressive primary aphasia patients who showed FDG-PET allowed researchers to detect abnormalities in the early stage of the disease (31). Our results also showed that the abnormal metabolism was associated with the disease status, which was consistent with the previous studies (12, 13, 31). This study was also the first quantitative descriptive FDG-PET study to certify that FDG-PET abnormal metabolism decreased following the prolongation of the disease duration. Therefore, the positive rate of semi-quantitative analysis may be higher than the present result if there are no significant disease duration difference among the patients.

Limitations of this study include the retrospective design, the significant different disease duration among the patients and relative small sample size of normal metabolism by visual analysis. Because abnormal metabolism was associated with different disease status, this will lead to misunderstandings of abnormal status. Although the whole sample size of AE was large, the patients with normal metabolism by visual analysis were relatively few, this need be evaluated by enrolling more patients in the future. Moreover, the patients were enrolled at a specialized tertiary epilepsy center, making the study subject to referral bias.

This study which showed semi-quantitative brain FDG-PET analysis was better than visual analysis in view of observing the abnormal glucose metabolism of patients with antibody positive AE. Semi-quantitative FDG-PET analysis appears to be a helpful tool in early diagnosis of patients with AE, especially those with normal MRI scans. Further research is needed to validate the sensitivity and specificity of semi-quantitative FDG-PET analysis in the early diagnosis of patients with AE. Besides, its usefulness for a better characterization of specific syndromes and their clinical course and response to therapy also needs to be further evaluated in prospective studies.

## Ethics Statement

Informed consent to participate the study and for publication for clinical details were obtained from each subject enrolled. The study was approved by the Medical Ethics Committee of Beijing Tiantan Hospital, Capital Medical University and was carried out in accordance with the Declaration of Helsinki.

## Author Contributions

R-JL and JL contributed to the study concept and design, and critically revised the manuscript for important intellectual content. R-JL, X-QS, QW, and X-BZ acquired the data. JP, GZ, JL, and R-JL analyzed and interpreted the data. R-JL, JP, and JL drafted the manuscript and provided statistical expertise. QW and X-QS provided administrative, technical, and material support. X-BZ and JL supervised the study.

### Conflict of Interest Statement

The authors declare that the research was conducted in the absence of any commercial or financial relationships that could be construed as a potential conflict of interest.
